# Global climate change and *Macadamia* habitat suitability: MaxEnt-based prediction under future scenarios

**DOI:** 10.3389/fpls.2025.1658566

**Published:** 2025-09-11

**Authors:** Yifan Li, Tao Zhong, Ya Ning, Yuchun Chen, Tingmei Yang, Hai Yue, Yaowen Yang, Hong Zhao, Haibin Wu, Zhaoqiang Jin, Jin Liu

**Affiliations:** ^1^ Yunnan Institute of Tropical Crops, Xishuangbanna, China; ^2^ Yunnan Key Laboratory of Sustainable Utilization Research on Rubber Tree, Xishuangbanna, China; ^3^ College of Agriculture, Yangtze University, Jingzhou, Hubei, China

**Keywords:** macadamia nuts, MaxEnt, climate change, ecological distribution, habitat change

## Abstract

Global climate change poses a major challenge for contemporary forestry. *Macadamia* is an economically valuable tree genus that is widely cultivated across multiple countries and regions. However, few studies have focused on its adaptive distribution and spatiotemporal dynamics under projected global warming scenarios. In this study, we collected the global occurrence records of two commercial *Macadamia* species (*Macadamia integrifolia* Maiden & Betche and *Macadamia tetraphylla* L.A.S. Johnson) and employed a parameter-optimized MaxEnt model to project their suitable habitats under current and future climate scenarios. The optimized model exhibited excellent predictive performance (AUC = 0.979), with a regularization multiplier of 0.5 and linear–quadratic–hinge feature combination. Key bioclimatic variables include: annual Mean temperature (bio1), isothermality (bio3), min temperature of coldest month (bio6), annual precipitation (bio12), and precipitation of driest month (bio14), which collectively comprise 88.2% of the model’s explanatory power. Under the current scenario, the most suitable cultivation areas were determined to be located in Australia, China, South Africa, Brazil, Madagascar, Argentina, and the United States. Compared with the current scenario, total habitat areas under future scenarios (specifically SSP126/585 in the 2030s and 2050s; SSP126/245/370 in the 2070s) are projected to increase by 1.13–7.51%, while reductions of 0.03–2.98% are projected under the other scenarios (SSP245/370 in the 2030s and 2050s; SSP585 in the 2070s). Notably, Brazil exhibits habitat reductions of 2.59–20.06% across all scenarios, while China shows increases of 0.70–45.11%. Furthermore, *M. integrifolia* was determined to exhibit greater cultivation potential and global expansion feasibility in range than *M. tetraphylla*. This study elucidates the dominant environmental drivers, current habitat suitability, and climate-driven shifts in *Macadamia* distribution, providing an empirical basis for sustainable cultivation under climate change.

## 1 Introduction

Species distributions are dramatically shaped by climatic shifts, with accumulating evidence indicating they have profoundly disrupted global biogeographic patterns (Jochum et al., 2007; Doxford and Freckleton, 2012; Matías et al., 2017; Zhao et al., 2021). The IPCC Fifth Assessment Report projects persistent global warming through 2100, predicting a temperature increase of 0.3–4.8°C relative to the 1986–2005 reference period (IPCC, 2013). Consequently, studying the potential geographic distribution of species under future climate change scenarios has become a critical research focus in numerous fields, including ecology, forestry, and agronomy (Zhao et al., 2024b; Wang et al., 2025; Zhang et al., 2024; Zhao et al., 2024a).

Species distribution models (SDMs), also known as ecological niche models (ENMs), are computational tools that integrate species occurrence data with environmental variables to predict biogeographic shifts under different environmental conditions, e.g., climate change scenarios (Wiens et al., 2009). SDMs are extensively employed to reconstruct historical biogeographic patterns and forecast future distributional dynamics (Wang et al., 2020). Common SDMs include generalized linear models (Nelder and Wedderburn, 1972), generalized additive models (Hastie and Tibshirani, 1986), genetic algorithm for rule-set production (Su et al., 2021), Maximum Entropy (MaxEnt) (Phillips et al., 2006), bioclim (Booth et al., 2014), and the land administration domain model (Lemmen et al., 2015). Among these models, MaxEnt has been widely used in ecological research owing to its user-friendly interface, computational efficiency, and reliable performance with small sample sizes (Lu et al., 2024).

The genus *Macadamia* includes two commercially cultivated species, *Macadamia integrifolia* Maiden & Betche and *Macadamia tetraphylla* L.A.S. Johnson. Both species are native to subtropical rainforests along the eastern coastline of Queensland and northern New South Wales in Australia (Nagao et al., 1992). Through selective breeding and adaptive cultivation, these species are now widely grown in Hawaii, South Africa, and China (Moncur et al., 1985; INC, 2023). According to the Yunnan Forestry and Grassland Bureau (Yunnan Forestry and Grassland Bureau, 2022), the global macadamia nut cultivation area reached approximately 4.67 million km^2^ in 2021, with China accounting for 54% of this area (2.53 million km^2^). This suggests that there is substantial potential for further expanding cultivation areas. We hypothesized that climate change may drastically alter macadamia nut cultivation distribution and productivity, with profound implications for global production. Therefore, predicting shifts in suitable habitats under future climates is crucial for strategic planning.

While numerous studies have explored the broader impacts of climate change on agricultural productivity and species distributions (Duan et al., 2022; Kong et al., 2021; Lu et al., 2024), research specifically addressing the habitat suitability for macadamia nut under changing climate conditions remains limited. To date, the only published national-level analysis quantifying climate suitability impacts was conducted in Malawi (Zuza et al., 2021). To address this research gap, this study used the MaxEnt model to predict how climate change will impact the distribution of suitable areas for the two *Macadamia* species. By modeling the species both jointly and separately, we will analyze the effects of climate change on global macadamia nut cultivation and compare the spatial distribution patterns of the two species. This analysis will provide critical insights for sustainable cultivation practices and industry planning under climate change.

## 2 Materials and methods

### 2.1 Cultivable *Macadamia* distribution data

Occurrence data for two commercial *Macadamia* species (*Macadamia integrifolia* Maiden & Betche and *Macadamia tetraphylla* L.A.S. Johnson) were collected through a comprehensive review of peer-reviewed literature and research platform records. A total of 2,948 occurrence records (1083 for *M. integrifolia*, 1865 for *M. tetraphylla*) were compiled from the Global Biodiversity Information Facility (GBIF; https://www.gbif.org/, accessed March 2025), peer-reviewed literature (Pei et al., 2025; Li et al., 2024a; Duan et al., 2025; Zhou et al., 2025), field surveys, and the Chinese Virtual Herbarium (CVH, https://www.cvh.ac.cn/). The data collected covers the period from 1850 to 2025. Georeferenced occurrences were clustered in eastern Australia, southern China, and eastern Africa, consistent with the known native and cultivated ranges of the species. For records with locality descriptions but no coordinates, latitude and longitude were determined using Google Maps. Duplicate or erroneous records were excluded from the study.

### 2.2 Environment variables

Data on 19 bioclimatic variables (**Supplementary Table S1**) and elevation at 2.5 arc-minute resolution were downloaded from WorldClim v2.1 (http://www.worldclim.org/) as GeoTIFF files. The slope and aspect were derived from elevation data using ArcGIS 10.8 (Esri, Redlands, CA, USA). The current climate scenario was adopted as the average for the years 1970-2000. Future climate projections were based on four Shared Socio-economic Pathways (SSP126, SSP245, SSP370, and SSP585) from the Coupled Model Intercomparison Project Phase 6 (CMIP6), covering three periods (2030s, 2021–2040; 2050s, 2041–2060; 2070s, 2061–2080). The BCC-CSM2-MR global climate model (GCM) developed by the National Climate Center was selected for analysis.

### 2.3 Environmental variable screening and model optimization

**Figure 1:**
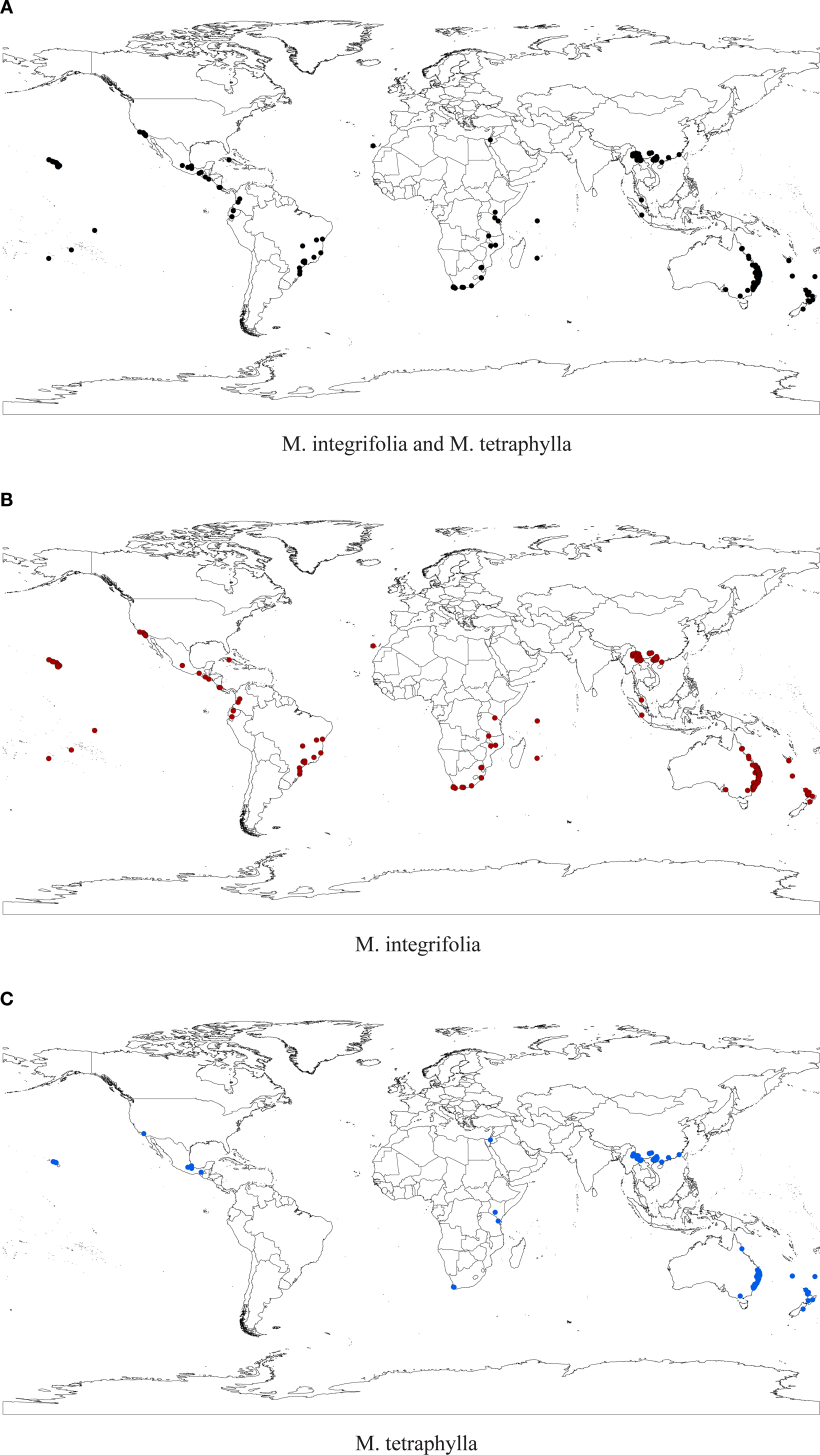
Distribution points of *M. integrifolia* & *M. tetraphylla* **(a)**, *M. integrifolia* **(b)**, and *M. tetraphylla* **(c)** after screening with ENMTools.

To mitigate potential model overfitting caused by spatial autocorrelation among sample points, occurrence records for both *Macadamia* species underwent redundancy screening using the R package ENMtools. This process resulted in 559 records for the joint species distribution model, 353 records for the *M. integrifolia* model, and 308 records for the *M. tetraphylla* model (**Figure 1**). The MaxEnt software requires input files in CSV format containing the following three columns: species name, longitude, and latitude. Environmental variables were extracted for each occurrence record according to their geographic coordinates. To mitigate multicollinearity, we eliminated environmental variables with strong correlations (i.e., based on the absolute value of Pearson’s correlation coefficient, |r| > 0.75) using SPSS version 25.0 (IBM Corp., Armonk, NY, USA) (**Supplementary Figure S1**). Finally, 12 environmental variables were used in the subsequent model analysis (**Supplementary Table S2**). Using ArcGIS 10.8, we converted the environmental layers into ASCII format before importing them, along with the filtered occurrence records, into MaxEnt version 3.4.4 for modeling.

We obtained MaxEnt version 3.4.4 from the official website (https://biodiversityinformatics.amnh.org/open_source/maxent/). MaxEnt incorporates five feature classes: linear (L), quadratic (Q), hinge (H), product (P), and threshold (T). By default, the regularization multiplier (RM) was set to 1 in MaxEnt. Feature combinations were selected based on species occurrence records: linear features were always included; quadratic features were applied for >10 points; hinge features for >15 points; and threshold and product features for >80 points (Elith et al., 2011). The R package ENMeval (Zhao et al., 2024c) was used to test the modified Akaike Information Criterion (AICc) values (i.e. AIC) of the MaxEnt model for different features (L, LQ, H, LQH, LQHP, and LQHPT) and different RM values (0.5–6, with 0.5 intervals for each parameter) parameter conditions. AIC was used to assess the fit and complexity of the model, and the difference between the training and testing AUC values (AUC.DIFF) and the 10% training omission rate (OR 10) were used to assess the degree of overfitting of the model (Zhao et al., 2021). The parameter configuration that yielded the lowest AIC value was selected to construct the final model. The model was calibrated using ten replicates under a 75:25 split for the training and testing datasets to ensure robust validation. The maximum number of iterations is set to 10000, and the convergence threshold is set to 0.00001.

### 2.4 Delineation of suitable habitats

Habitat suitability probabilities under current and future climate scenarios were classified into discrete categories using the Jenks natural breaks algorithm, minimizing the within-class variance. The resulting habitat suitability categories were defined as follows: unsuitable habitat (<0.03 probability of occurrence), lowly suitable habitat (0.03–0.24), moderately suitable habitat (0.24–0.41), and highly suitable habitat (>0.41). ArcGIS 10.8 was used to reclassify the ASCII files generated after the model was run, and the areas of highly, moderately, and lowly suitable habitats of macadamia nut were calculated.

### 2.5 Contraction and expansion of suitable habitats

This study used maximum training sensitivity plus specificity (MTSPS) as the logical threshold to analyze changes in the spatial pattern of *Macadamia* suitability areas. The model-generated layers were converted to binary layers using ArcGIS 10.8. The logical value of L ≥ 0.13 was used to define the potential habitats and assigned as ‘1’, while the logical value of L < 0.13 was defined as unsuitable habitats and assigned as ‘0’. The changes in the habitat areas of the species were classified into four types in this study: unsuitable, range contraction, no change, and range expansion. The future distributional expansion/contraction of *Macadamia* was compared with the potential habitats for the species under current climate scenarios. The value of the binary layer for current suitable habitats was multiplied by two and then subtracted the binary layer value for future suitable habitats. The resulting values after subtraction were interpreted as follows: 0 (no occupancy), -1 (range expansion), 1 (no change), and 2 (range contraction).

## 3 Results

### 3.1 Model optimization and evaluation

**Figure 2:**
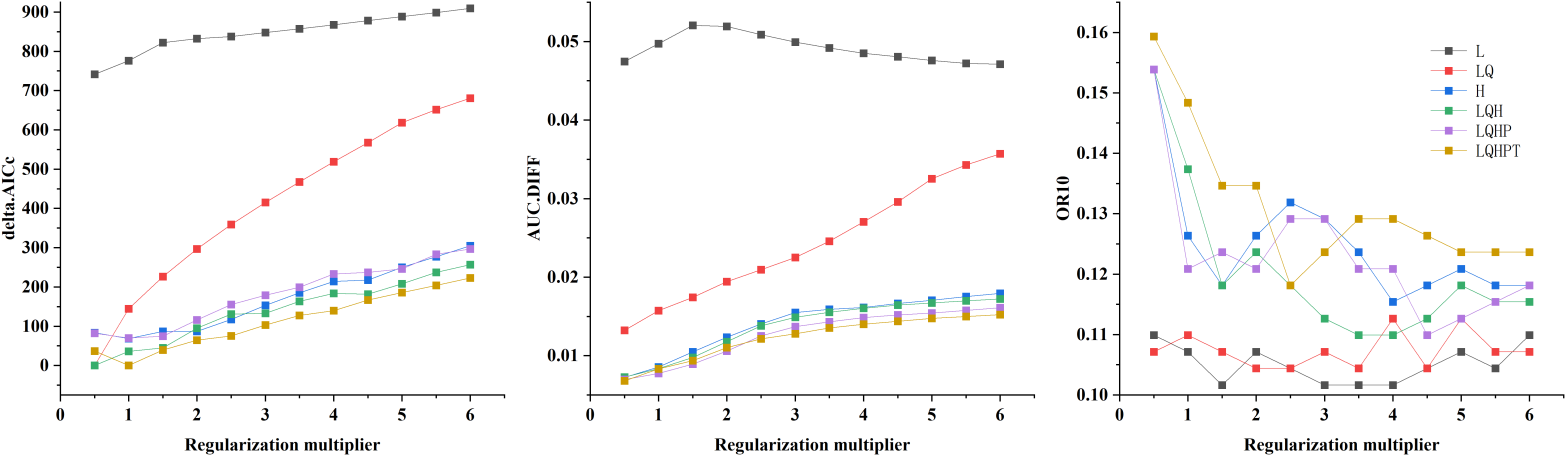
Model evaluation indicators generated by ENMeval.

**Figure 3:**
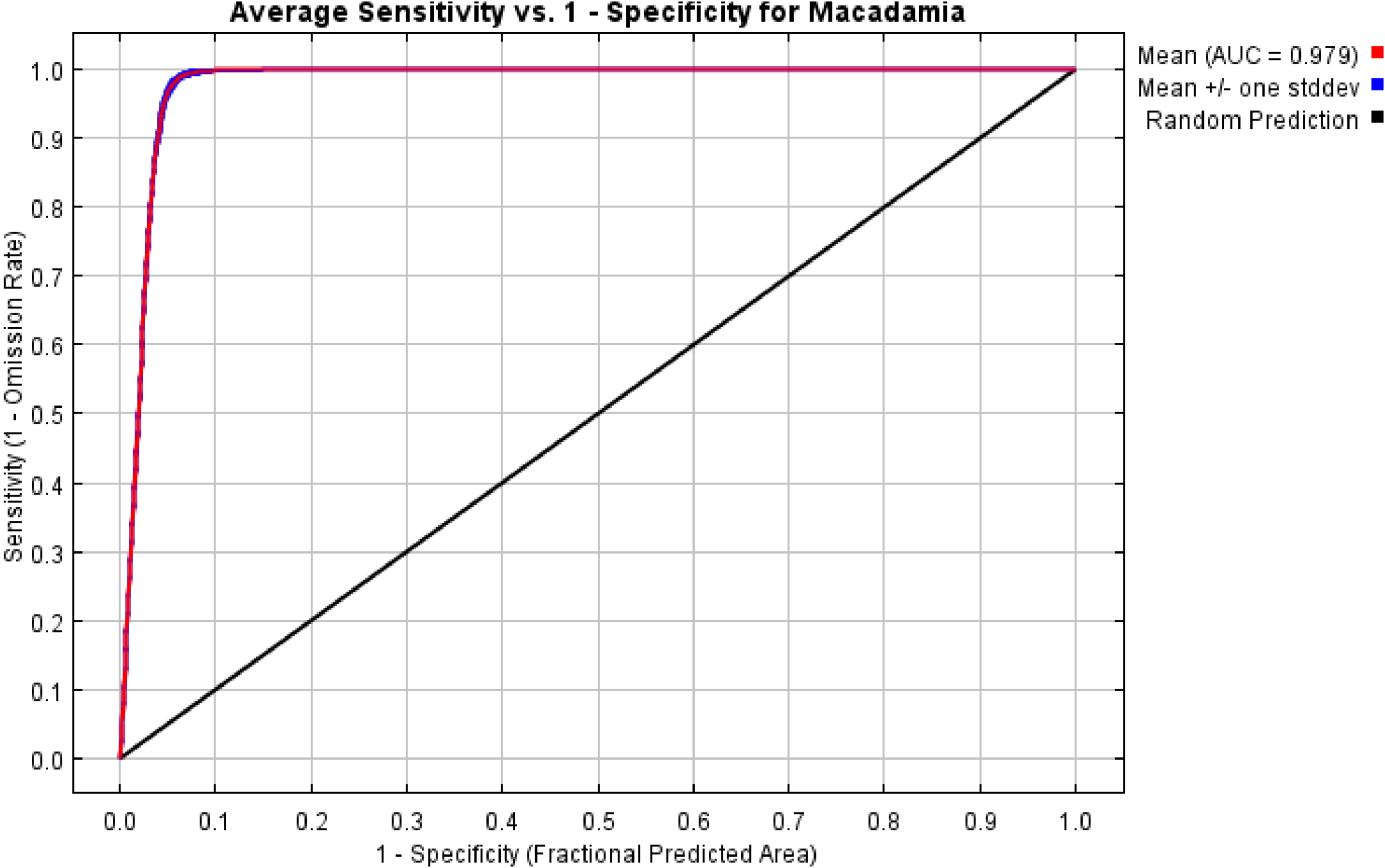
Receiver operating characteristic (ROC) curve and area under the curve (AUC) values of the MaxEnt model.

In this study, we systematically evaluated the effects of 12 regularization multipliers (RM) and 6 feature combinations (FC) on model performance. As shown in **Figure 2**, the lowest AICc value occurred under two configurations: RM = 0.5 with FC = LQH, and RM = 1 with FC = LQHPT. The RM = 0.5/LQH combination yielded a lower AUC.DIFF and higher OR10 than the alternative. Considering model generalization capability and omission rates, we ultimately selected RM = 0.5 and FC = LQH. Parameter optimization resulted in an average AUC of 0.979 over 10 model runs, confirming the MaxEnt model’s reliability for predicting the global ecological distribution of *Macadamia* habitats (**Figure 3**).

### 3.2 Evaluation of MaxEnt model prediction results

**Figure 4:**
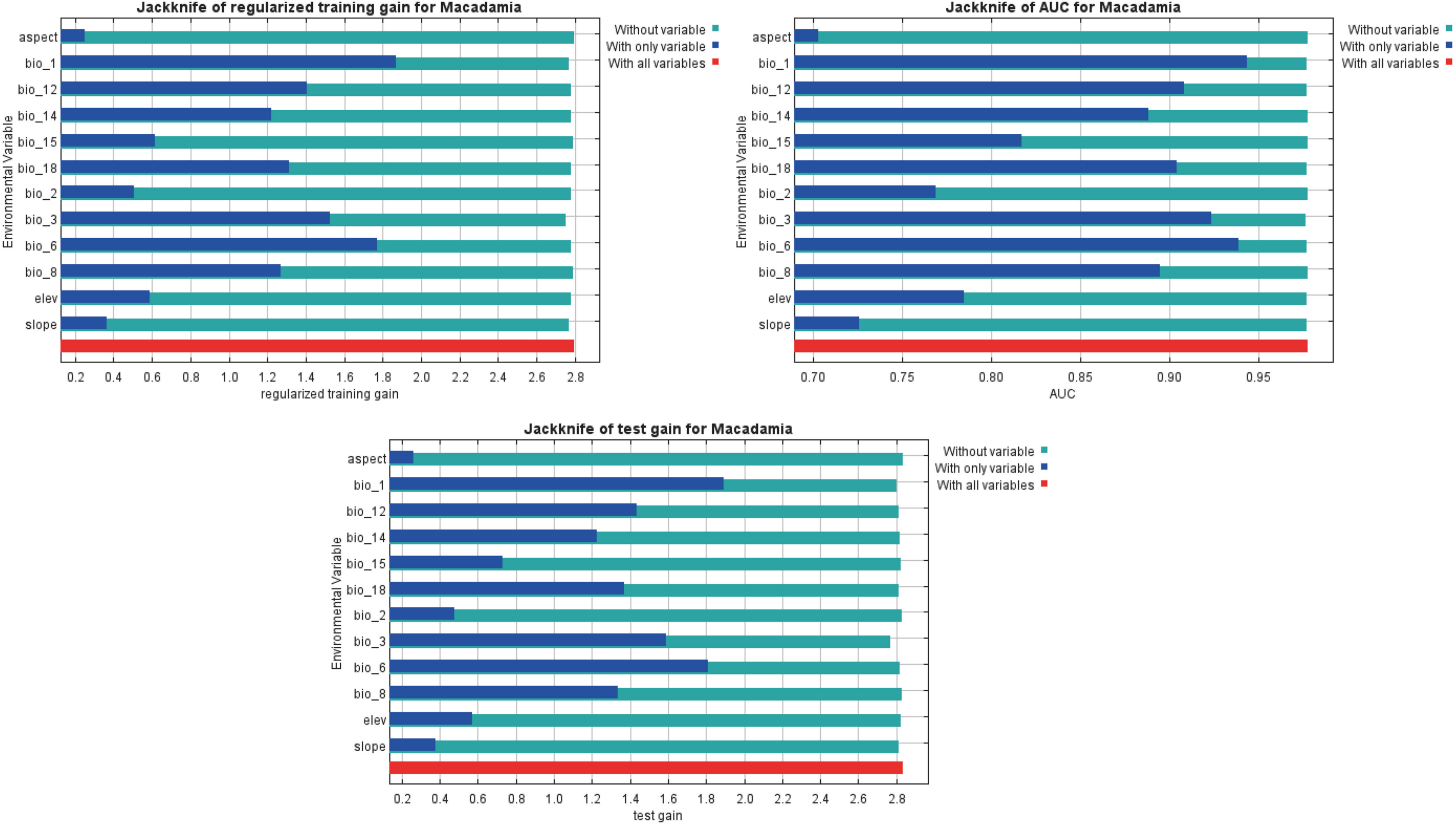
Jackknife test of the optimized MaxEnt model.

The jackknife test generated by MaxEnt reflected the weights of the effects of different environmental factors on habitat suitability. As shown in **Figure 4**, the only variable test (blue bars) revealed that the top three bioclimatic variables in terms of regularized training gain, AUC, and test gain were annual mean temperature (bio1), isothermality (bio3), min temperature of coldest month (bio6). Thus, these three bioclimatic variables had obvious effects on the *Macadamia* distribution. The without-variable test (green bars) showed that bio3 exhibited the most pronounced decrease in regularized training gain, AUC, and test gain. This finding suggests that bio3 contains more specific environmental information, which is useful for predicting the distribution of macadamia nut. Additionally, the contributions of individual bioclimatic variables to the MaxEnt model are presented in **Supplementary Table S2**. The five most influential factors were bio3 (30.0%), bio12 (20.0%), bio14 (19.6%), bio1 (10.1%), and bio6 (8.5%). Together, these five factors accounted for 88.2% of the total contribution of variables to the optimized MaxEnt model. The permutation importance values for these five factors were 10.6% (bio3), 7.9% (bio12), 3.8% (bio14), 22.4% (bio1), and 42.2% (bio6), totaling 86.9%. These results indicate that these five environmental variables are the key determinants of *Macadamia* distribution.

**Figure 5:**
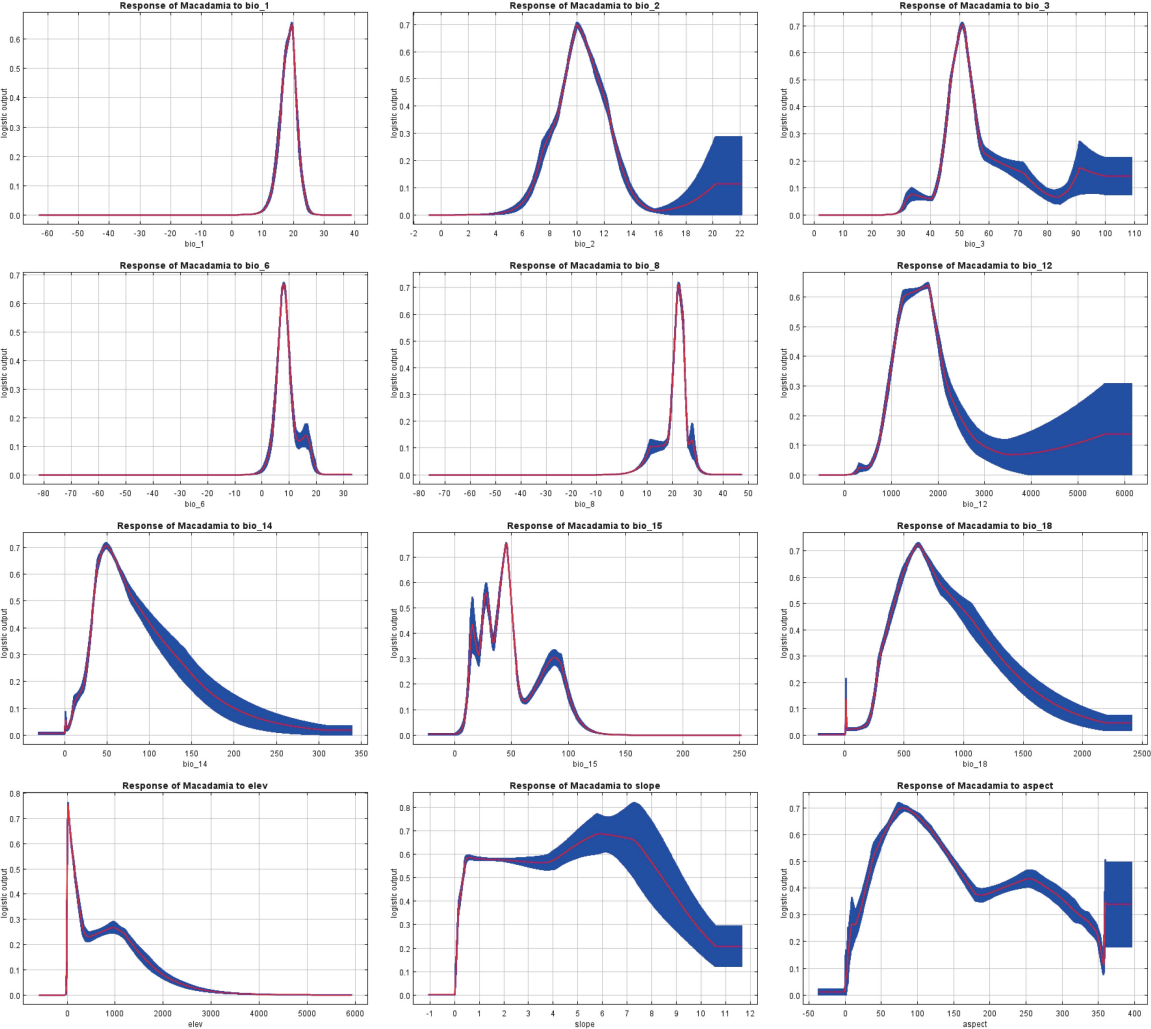
Response curves of the 12 environmental variables.

Response curves show the quantitative relationship between environmental variables and the logistic probability of presence (i.e., habitat suitability). The MaxEnt model simulates a species distribution, and its response curves (**Figure 5**) effectively estimate the range of suitable habitats. The presence probability threshold (L > 0.41) was used to identify the environmental conditions that are highly suitable for macadamia nut cultivation. The optimal ranges of environmental variables were as follows: annual mean temperature (bio1, 16.25–20.84°C), mean diurnal range (bio2, 8.75–12.18°C), isothermality (bio3, 46.01–55.03%), min temperature of coldest month (bio6, 5.77–10.03°C), mean temperature of wettest quarter (bio8, 20.34–24.80°C), and annual precipitation (bio12, 1043.46–2055.65 mm). Additionally, precipitation of driest month (bio14, 31.29–103.00 mm), while precipitation seasonality (bio15) showed discontinuous ranges (14.47–16.66, 23.53–32.04, and 35.89–51.54). Precipitation in warmest season (bio18, 359.49–1122.30 mm). Topographic variables included elevation (5.49–207.56 m), slope (0.21–9.06°), and aspect (29.18–172.56° and 222.37–276.95°).

### 3.3 Global distribution of *Macadamia* suitable habitats under the current climate scenario

**Figure 6:**
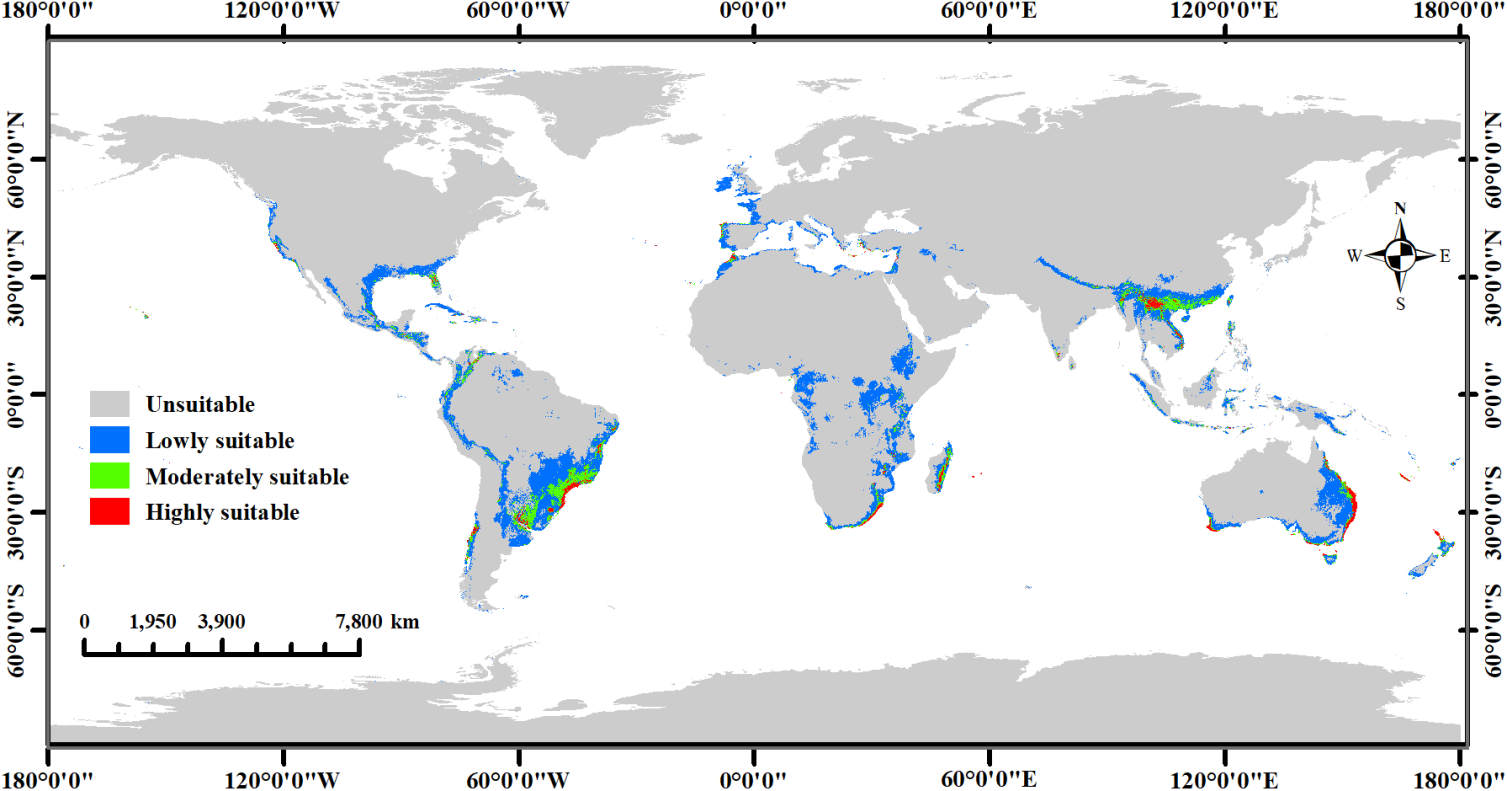
Global ecological distribution of *Macadamia* under current climate scenarios.

The MaxEnt model predicted the potential global distribution of two commercial *Macadamia* species under the current climate scenario (**Figure 6**). Highly suitable habitats are primarily located along the eastern, western, and southern coasts of Australia, southwestern China, southeastern South Africa, southeastern Brazil, eastern Madagascar, northeastern Argentina, and the southeastern and western coasts of the United States. Additionally, New Zealand, Portugal, Spain, Greece, Turkey, Syria, Lebanon, India, Sri Lanka, Nepal, Bhutan, Vietnam, Myanmar, Laos, Indonesia, the Philippines, New Caledonia (France), Réunion (France), Fiji, Morocco, Malawi, Tanzania, Mozambique, Zimbabwe, Mauritius, Mexico, Cuba, Jamaica, Honduras, Guatemala, Venezuela, Colombia, Uruguay, and Chile contain limited areas of highly suitable habitats. Moderately suitable habitats were typically adjacent to highly suitable areas. Consequently, countries containing highly suitable habitats generally also possessed moderately suitable areas. However, some countries have only moderately suitable habitats, such as Kenya, the Republic of the Congo, Eswatini, Malaysia, Brunei Darussalam, Papua New Guinea, Nicaragua, Costa Rica, Panama, the Dominican Republic, Haiti, Peru, Bolivia, Paraguay, and Cyprus. The distribution of lowly suitable habitats is extensive, with large areas present in central Africa, central South America, southern North America, western Europe, southern Asia and its associated archipelagos, and eastern Oceania and its associated archipelagos. Overall, the area of highly suitable habitats was 73.35 × 10^4^ km^2^ (5.51% of the total habitat area), while the area of moderately and lowly suitable habitats accounted for 193.45 × 10^4^ km^2^ (14.54%) and 1063.37 × 10^4^ km^2^ (79.94%), respectively (**Table 1**).

**Table 1:** Comparison of different *Macadamia* suitable habitats under four future climate scenarios (SSP126, SSP245, SSP370, and SSP585) with that under the current climate scenario.

Species	Climate scenario		Lowly suitable habitats (×10^4^ km^2^)	Moderately suitable habitats (×10^4^ km^2^)	Highly suitable habitats (×10^4^ km^2^)	Total suitable habitats (×10^4^ km^2^)
*Macadamia integrifolia* Maiden & Betche, and *Macadamia tetraphylla* L.A.S.Johnson		Current	1063.37	193.45	73.35	1330.17
	SSP126	2030	1095.46 (3.02)	207.91 (7.47)	85.62 (16.73)	1389.09 (4.43)
		2050	1089.97 (2.50)	169.97 (−12.14)	69.68 (−5.00)	1329.76 (−0.03)
		2070	1069.16 (0.54)	185.45 (−4.14)	54.62 (−25.53)	1309.27 (−1.57)
	SSP245	2030	1147.51 (7.91)	204.00 (5.45)	78.38 (6.86)	1430.01 (7.51)
		2050	1063.38 (0.00)	204.65 (5.79)	77.11 (5.14)	1345.21 (1.13)
		2070	1021.84 (−3.91)	208.59 (7.82)	83.90 (14.38)	1314.44 (−1.18)
	SSP370	2030	1019.52 (−4.12)	194.78 (0.68)	76.24 (3.95)	1290.59 (−2.98)
		2050	1132.99 (6.55)	205.70 (6.33)	78.69 (7.29)	1417.52 (6.57)
		2070	1116.61 (5.01)	161.78 (−16.37)	73.05 (−0.40)	1351.66 (1.62)
	SSP585	2030	1138.85 (7.10)	192.91 (−0.28)	72.07 (−1.73)	1403.90 (5.54)
		2050	1091.60 (2.65)	216.94 (12.14)	84.86 (15.70)	1393.55 (4.76)
		2070	1041.56 (−2.05)	192.76 (−0.36)	71.21 (−2.91)	1305.56 (−1.85)

The values in parentheses indicate the percentage by which the area of suitable habitats under the climate scenario is higher or lower (–) than that under the current climate scenario.

**Figure 7:**
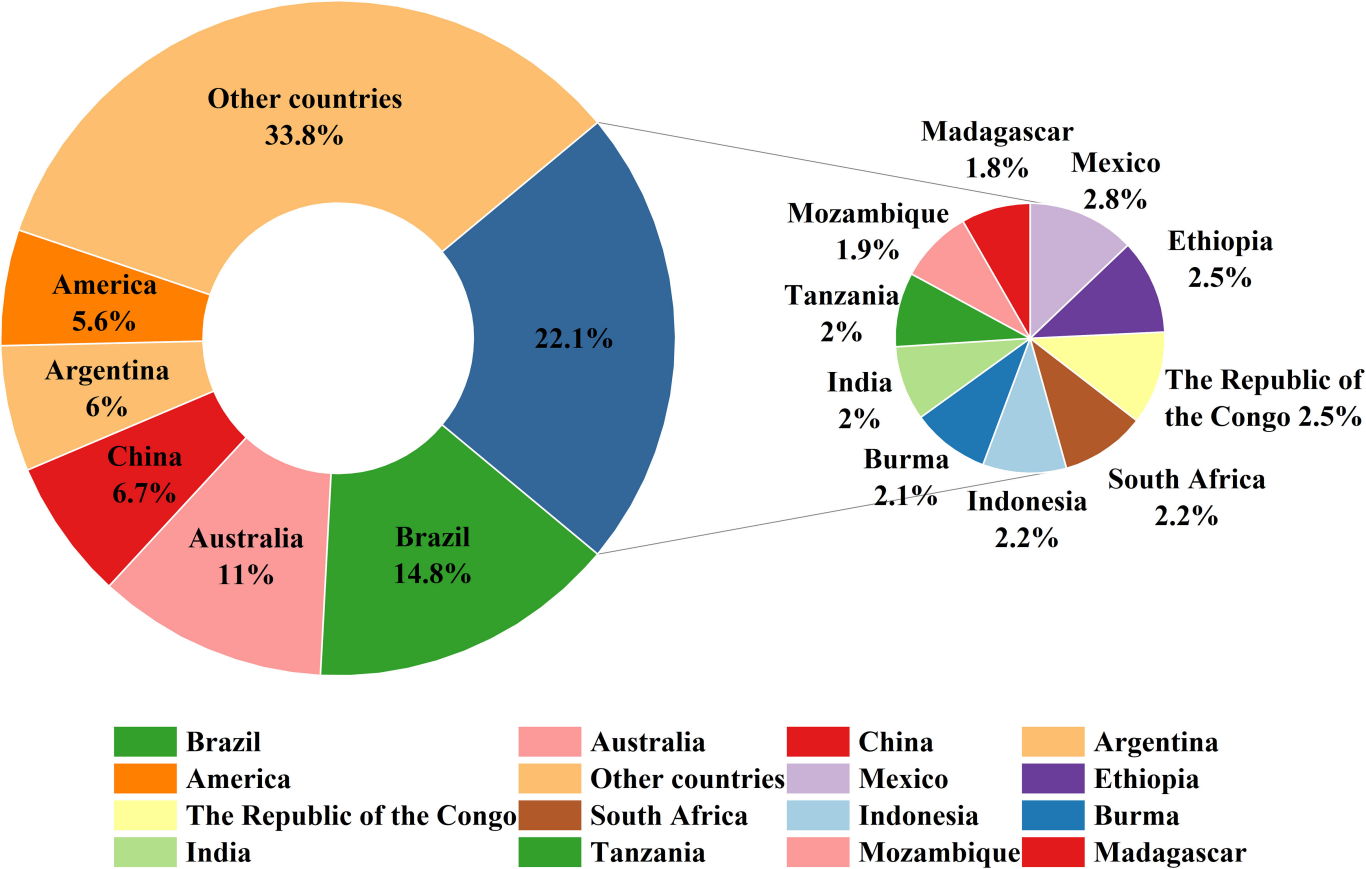
Top 15 countries and their share of global *Macadamia* suitable habitats under the current climate scenario.

In addition, to further analyze the distribution patterns at national scales, we classified the predicted suitable habitats by country and calculated their respective areas. **Figure 7** shows that Brazil possessed the largest total suitable area (14.8% of global suitable habitats), followed by Australia (11%), China (6.7%), Argentina (6%), and the United States (5.6%). Other countries with significant suitable areas included Mexico, Ethiopia, the Republic of Congo, South Africa, Indonesia, Myanmar, India, Tanzania, Mozambique, and Madagascar.

### 3.4 Global suitable habitat distribution for *Macadamia* under future climate scenarios

The present study considered four climate scenarios (SSP126, SSP245, SSP370, and SSP585) across three future periods (2030s, 2050s, and 2070s) to predict the global distribution of suitable habitats for *Macadamia* (**Supplementary Figure S2**). The distribution of suitable habitats for *Macadamia* was significantly altered under the various climate scenarios across the different future periods (**Table 1**).

Under the SSP126 scenario, potential *Macadamia* habitat areas in China were predicted to increase substantially across all three future periods, whereas Brazil and parts of Western Europe projected significant decreases. Additionally, south-central Africa showed substantial habitat area increase in the 2030s and 2050s, whereas Australia exhibited a significant decrease during these periods. In the 2070s, the projected decrease in potential suitable area for south-central Africa is expected to exceed the increase, while Australia is expected to undergo a dramatic increase. Overall, the total suitable habitat area increased significantly across all three periods, with increases of 1.13% to 4.43%. The increase in total suitable habitat area in the 2030s and 2050s was primarily associated with the lack of decrease in lowly, moderately, and highly suitable habitats. In the 2070s, the increase in total suitable habitat area was mainly driven by gains in lowly suitable habitats, which outweighed the concurrent losses in moderately and highly suitable habitats.

Under the SSP245 scenario, potential *Macadamia* habitats were projected to increase substantially in China, Mexico, and Australia across all future periods, whereas those in Brazil, Western Europe and south-central Africa decreased significantly. Overall, the total habitat area decreased in the 2030s and 2050s (contractions of 0.03−1.18%), whereas a significant increase (5.54%) occurred in the 2070s. The decrease in the 2030s was mainly driven by declines in moderately and highly suitable habitats, while in the 2050s, it was primarily associated with losses in lowly suitable habitats.

Under the SSP370 scenario, south-central Africa exhibited pronounced habitat increases and decreases across all periods. China and Mexico showed significant increases, while Brazil and Australia decreased substantially. Western Europe experienced marked decreases in the 2030s and 2050s, and the United States also decreased significantly in the 2030s. Overall, the total habitat area declined in the 2030s and 2050s (contractions of 1.57−2.98%) but increased by 4.76% in the 2070s. The predicted SSP370 decrease patterns in the 2030s and 2050s are consistent with those under the SSP245 scenario.

Under the SSP585 scenario, China exhibited substantial area increases across all periods. Brazil, south-central Africa, and Australia were projected to decrease significantly in the 2070s, while global habitat areas showed only minor and irregular decreases in the 2030s and 2050s. Overall, the total suitable habitat area increased in the 2030s and 2050s (increases of 6.57−7.51%), but decreased by 1.85% in the 2070s in association with declines across all habitat suitability levels.

**Figure 8:**
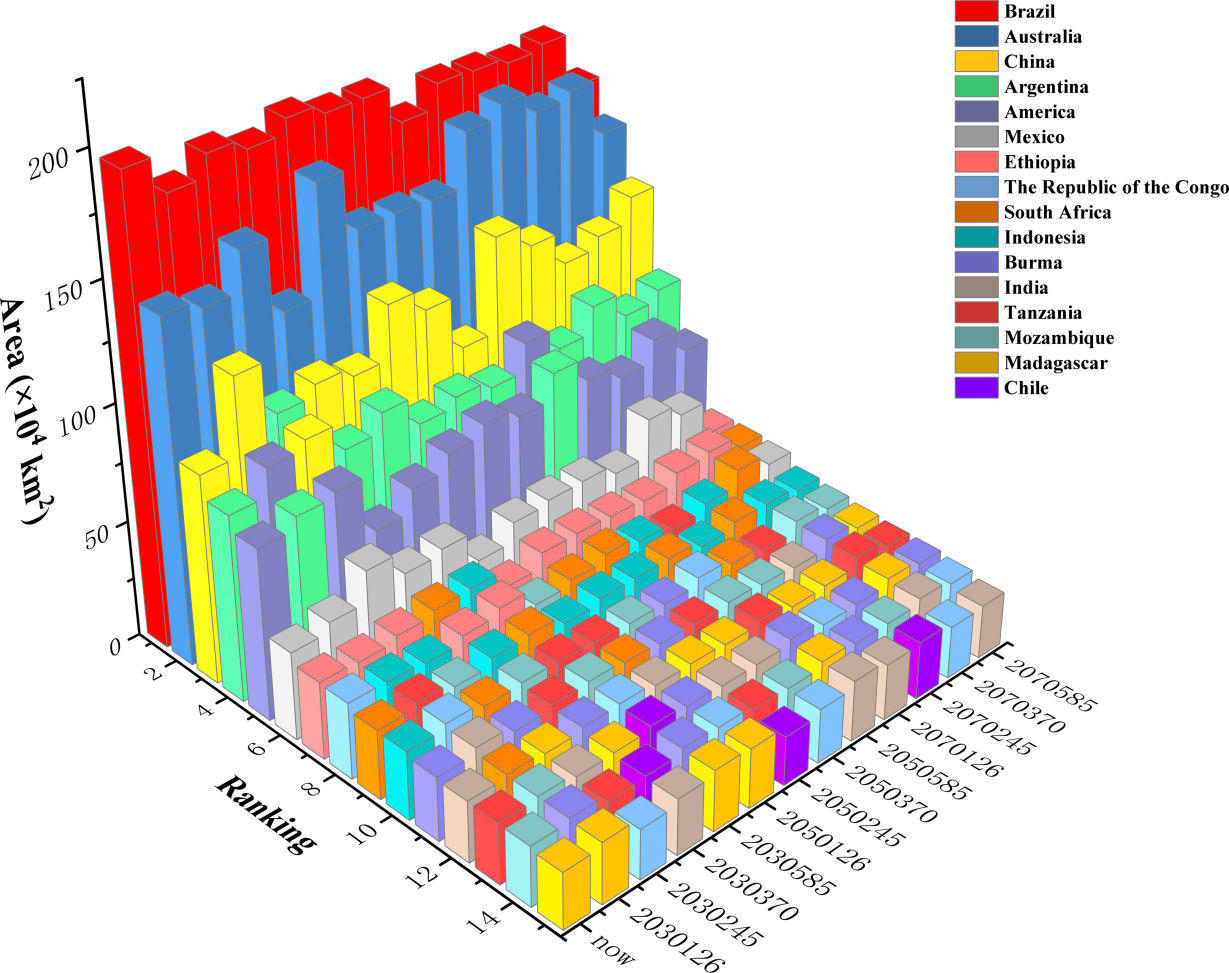
Total suitable habitat area for the top 15 countries under different climate scenarios.

At the national level, the top five countries with the largest suitable habitat areas across all climate scenarios were Brazil, Australia, China, Argentina, and the United States (**Figure 8**). Brazil consistently ranked first under all climate scenarios, although its total suitable habitat area decreased under future climate scenarios (ranging from 2.59% to 20.06%) compared to the current scenario. Australia maintained second place, but its suitable habitat area exhibited both increases and decreases under different climate scenarios: declines of 2.72% (2030s-SSP126), 13.23% (2030s-SSP370), 0.75% (2050s-SSP370), and 2.83% (2070s-SSP585) occurred, while increases of 0.15% to 17.67% occurred under other scenarios. China ranked third in suitable habitat area (except under the 2030s−SSP245 scenario), with consistent increases across all future climate scenarios (0.70% to 45.11%). Argentina generally ranked fourth (except under the 2030s−SSP126/245 and 2050s−SSP585 scenarios). Its suitable habitat area decreased under the 2030s−SSP126 and 2050s−SSP126/370 scenarios (by 1.61%, 1.48%, and 0.97%, respectively), while other scenarios showed significant increase (2.01% to 23.97%). The United States ranked fifth (except under the 2030s−SSP126 and 2050s−SSP585 scenarios), decreases of 23.69% (2030s-SSP370), 10.56% (2030s-SSP585), 0.90% (2050s-SSP126), 0.51% (2050s-SSP370), 8.04% (2070s-SSP245), and 11.18% (2070s-SSP585) occurred, while other scenarios exhibited increases of 0.81% to 24.09%. Countries ranked 6th to 15th included Mexico, Ethiopia, the Republic of Congo, South Africa, Indonesia, Myanmar, India, Tanzania, Mozambique, Madagascar, and Chile. Notably, Chile was not among the top 15 countries under the current climate scenario, but its suitable habitat area expanded significantly under all future scenarios.

In summary, potential *Macadamia* habitat area in Brazil was projected to decrease consistently across all scenarios, whereas China was projected to increase across all scenarios. Australia, Argentina, the United States, Mexico, south-central Africa, and Western Europe showed divergent responses to climatic changes, with both habitat gains and losses. These shifts indicate potential future disruptions in global macadamia nut production and associated supply chains.

### 3.5 Future dynamic changes in *Macadamia* habitats

**Figure 9:**
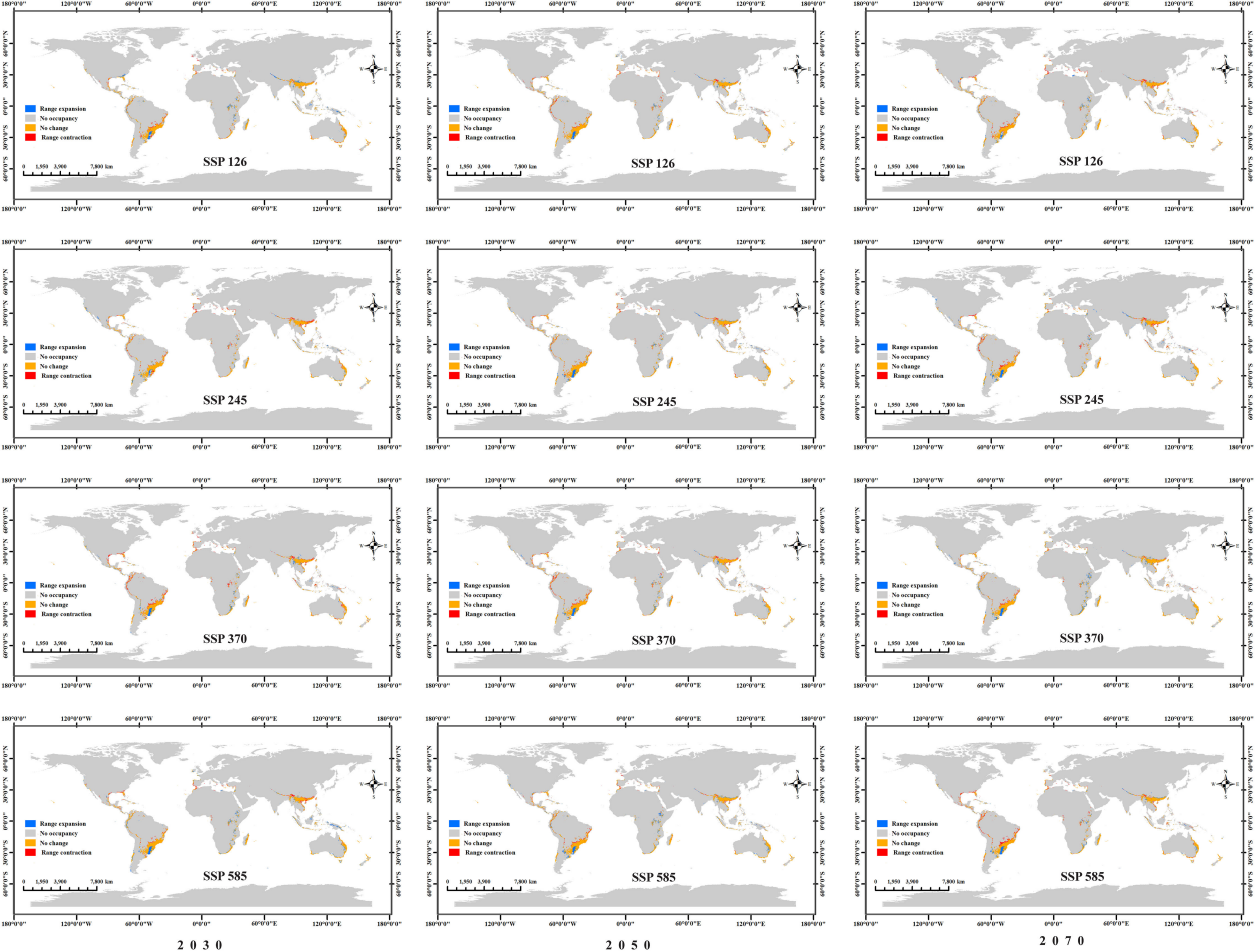
Expansion and contraction of potential *Macadamia* habitats under four future climate scenarios (SSP126, SSP245, SSP370, and SSP585) across three different periods (2030s, 2050s, and 2070s).

To quantitatively compare suitable habitat changes between current and future periods, this study applied the MTSPS as a threshold to assess the presence/absence of suitable habitat. As shown in **Figure 9**, relatively obvious contractions and expansions occur in central-southern South America and southern Asia, such as Brazil, Uruguay, Argentina, and Myanmar. Changes (expansions and contractions) in most other countries are sporadic. Overall, under the SSP126 and SSP585 scenarios, the expanded area decreases over time, while under the SSP245 and SSP370 scenarios, the expanded area increases over time. Under the SSP126 scenario, the contracted area increases over time, while under the SSP370 scenario, the contracted area decreases over time. No clear temporal pattern was observed for the contracted or expanded areas under the SSP245 and SSP585 scenarios (**Table 2**).

**Table 2:** Comparison of *Macadamia* contraction and expansion areas under different climate scenarios.

Climate scenario	Range expansion (× 10^4^ km^2^)	No change (× 10^4^ km^2^)	Range contraction (× 10^4^ km^2^)	Suitable area (× 10^4^ km^2^)
2030s-SSP126	106.86	535.24	61.75	642.11
2050s-SSP126	77.00	522.06	74.93	599.06
2070s-SSP126	72.18	506.53	90.46	578.71
2030s-SSP245	61.61	511.60	85.39	573.21
2050s-SSP245	84.96	527.72	69.27	612.68
2070s-SSP245	105.89	507.25	89.74	613.14
2030s-SSP370	77.30	477.22	119.77	554.51
2050s-SSP370	83.84	502.43	94.56	586.27
2070s-SSP370	118.07	536.07	60.92	654.14
2030s-SSP585	107.93	527.36	69.63	635.30
2050s-SSP585	104.91	537.80	59.19	642.72
2070s-SSP585	86.34	493.87	103.12	580.21

### 3.6 Comparison of potential habitats of two commercial *Macadamia* species

**Figure 10:**
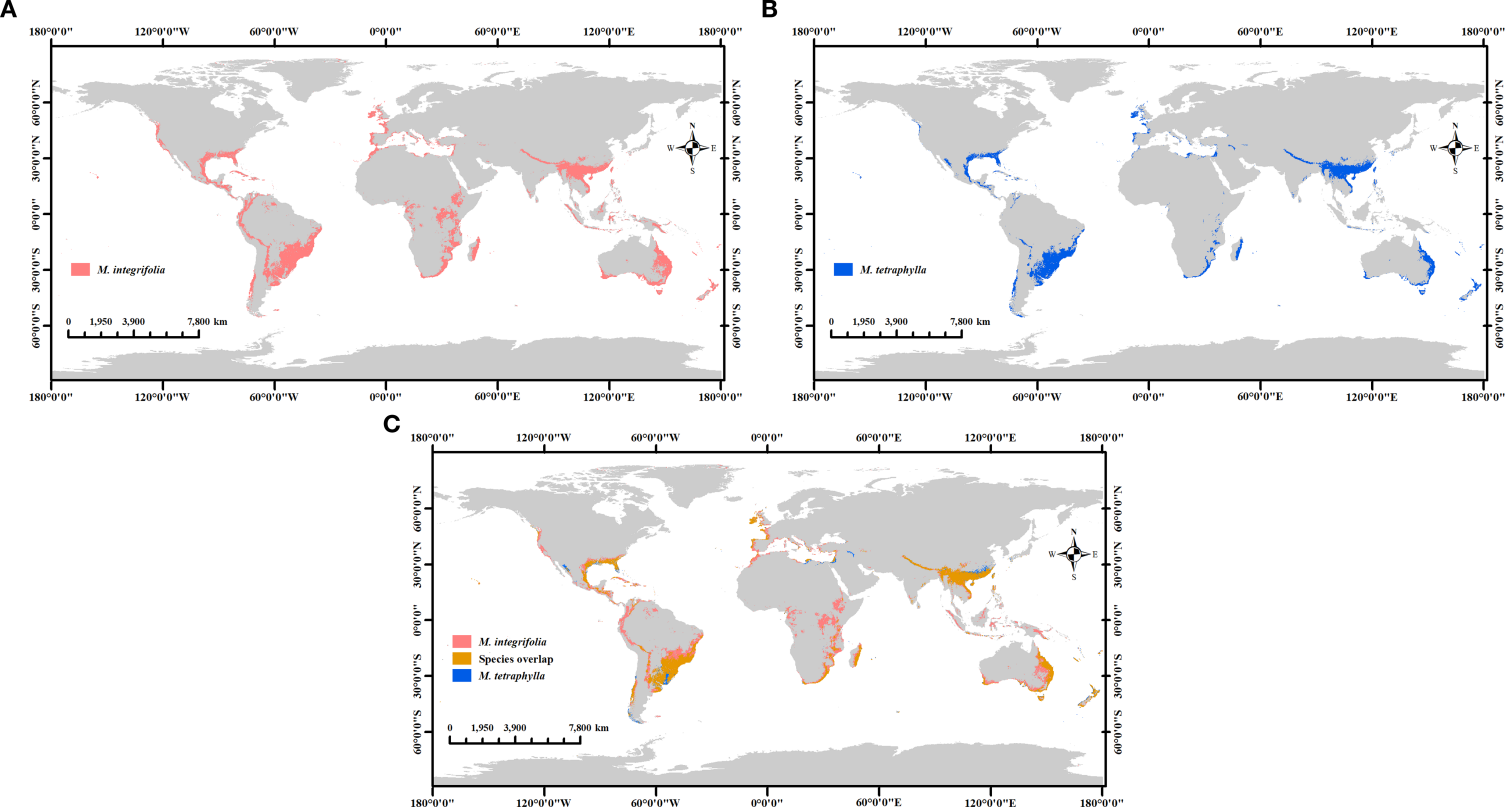
Comparing the distribution of potential habitats of two *Macadamia* species. *M. integrifolia* **(a)**, *M. tetraphylla* **(b)**, and *M. integrifolia* & *M. tetraphylla* overlap **(c)**.

The model parameters were set to operate in accordance with current climate scenarios, and the simulations were generated and analyzed using ArcGIS 10.8 software. As shown in **Figure 10**, *M. integrifolia* was determined to exhibit a significantly larger potential habitat area (1284.78 × 10^4^ km^2^) than that of *M. tetraphylla* (720.35 × 10^4^ km^2^). Notably, compared to *M. tetraphylla*, the distribution of *M. integrifolia* extends across a substantially larger area of potentially suitable habitat, including regions in northern and central South America, central Africa, Australia, the Malay Archipelago, the United States, Mexico, and parts of western Europe.

## 4 Discussion

### 4.1 Model optimization and evaluation

The model accuracy is critically influenced by the configuration of the feature parameters (Zhang et al., 2023). In this study, AICc, AUC.DIFF, and OR 10 were selected to evaluate the model’s fit and complexity. The results indicate that, when RM was set at 0.5 and FC was LQH, AICc was the lowest, suggesting that the model fits best under these two feature parameter settings. However, compared to RM was 1 and FC was LQHPT, when RM was 0.5 and FC was LQH, the model exhibits lower AUC.DIFF values and higher OR10 values (15.39% vs. 14.84%), indicating that this feature parameter setting confers stronger generalization ability but higher omission rates. It is widely accepted that an OR10 of 0.1 (10% omission) is generally acceptable (Gengping and Huijie, 2016). However, in this instance, the model’s capacity for fit and its generalization ability are of greater significance. A slight omission of more than 10% can be considered acceptable.

Predictive reliability and accuracy were evaluated using the area under the receiver operating characteristic curve (AUC) (Duan et al., 2022). Various ranges of AUC values were interpreted to indicate the following about the MaxEnt model discriminatory capacity: 0.50–0.59 (failure), 0.60–0.69 (poor), 0.70–0.79 (moderate), 0.80–0.89 (good), and ≥0.90 (excellent) (Kong et al., 2021; Zhang et al., 2021). AUC values approaching 1 indicated higher model discriminatory capacity. The mean AUC value across 10 replicates was 0.979, demonstrating the high reliability of the model in predicting the global distribution of *Macadamia* habitats.

### 4.2 Predicted results and actual environment

Temperature and precipitation are critical environmental factors affecting crop growth, yield formation, and geographic distribution (Jägermeyr et al., 2021; Lesk et al., 2021). Stephenson and Gallagher (1986) reported that growth rates of macadamia trees were the highest at 25–30°C. Moreover, kernels grown at 25°C consistently exhibited fat contents exceeding 72% at harvest. In contrast, lower kernel quality and growth were observed at 15°C and 35°C. A follow-up study by Stephenson and Gallagher (1987) found that five-year-old macadamia trees exhibited higher fruit drop rates at 30–35°C than at 20–25°C. Trochoulias and Lahav (1983) found that macadamia trees cultivated at 15–30°C for 80 d showed significant increases in leaf area, height, and stem thickness. However, plants cultivated at 10°C exhibited no growth, while those maintained at 30°C developed chlorophyll loss (yellowing) and necrosis. At 35°C, plants suffered from bud break proliferation and dehydration. Collectively, these studies indicate that temperature requirements of macadamia trees vary with growth stage, complicating the determination of a universal optimal range of factors.

Allan (1972) reported that the optimal average temperature for macadamia trees growth ranges between 23 and 24°C. Shi et al. (2016) further reported that macadamia trees exhibit optimal growth at temperatures of 20–25°C. However, our findings suggested that macadamia trees thrive at temperatures between 16.25 to 20.84°C. The model-predicted ranges were marginally lower than those reported by earlier studies, potentially due to regional or temporal constraints in prior research or the model’s inclusion of extreme cold events over the past three decades. Thus, the discrepancy between model predictions and historical observations does not suggest there were methodological errors. Additionally, the isothermality range (46.01−55.03%) suggests that macadamia trees exhibit adaptability to temperature fluctuations. However, the threshold for low temperature tolerance in macadamia trees remains underexplored. Li et al. (2009) investigated cold resistance in macadamia trees and reported semi-lethal temperatures between −0.72 and 2.36°C across 10 varieties. Jiang et al. (2000) found differential cold tolerance among cultivars: cultivars ‘344’ and ‘246’ showed no frost damage above −5°C, ‘788’ exhibited frost damage at −5°C, ‘800’ and ‘741’ at 0°C. Thus, the authors posited that regions with minimum temperatures above 0°C are optimal for macadamia nut cultivation. The concept of using minimum temperatures to identify suitable planting areas had been previously suggested (Shigeura, 1981b). According to Shigeura (1981a), *M. integrifolia* achieves optimal yield and fruit quality in areas with average minimum winter temperatures of 14−17°C, whereas *M. tetraphylla* actually prefers cooler conditions. Our model predicts the highly suitable high suitability for *Macadamia* cultivation in regions with coldest-month minimum temperatures of 5.77−10.03°C, slightly lower than the previously published recommendations.

Regarding water requirements, Li et al. (2024a) established that *Macadamia* grows optimally under an annual precipitation of 500–2000 mm. Our results support this conclusion, with viable cultivation determined by the present work to occur with 1043.46–2055.65 mm annual precipitation and dry-season minimum precipitation >31.29 mm. This indicates that the water requirements are generally high, yet adaptable to lower precipitation. Such adaptability may arise from soil moisture retention, enabling survival at ≤510 mm annual precipitation (Nagao et al., 1992). In addition, the geoenvironmental factors (elevation, aspect, and slope) further modulate plantation distribution via microclimatic effects. For example, sunny slopes experience higher temperatures owing to increased solar exposure, while windward slopes at the higher elevations typically receive greater rainfall. Trochoulias and Lahav (1983) suggested that macadamia nut cultivation is viable at up to 500 m above sea level, which is consistent with our inferred highly suitable habitats (5.49–207.56 m). However, in China, macadamia nut plantations are predominantly established at 800–1400 m (Li et al., 2024b), indicating their successful expansion into moderately/lowly suitable habitats under localized agroecological management. However, these comparisons highlight the inherent limitations of predictive results. Notwithstanding the optimization of the model, it remains incapable of circumventing the inherent complexity and uncertainty that are inextricably intertwined with predictive processes. Nevertheless, given the potential distribution and changes of species, model predictions still hold significant guiding significance and practical value for actual development.

### 4.3 Future changes in *Macadamia* distribution

Currently, the main macadamia nut producing countries worldwide are Australia, South Africa, China, Kenya, the United States, Guatemala, Brazil, New Zealand, Vietnam, Malawi, and Mozambique (Li et al., 2024a; INC, 2023), consistent with this study’s current-climate simulations. According to statistics, the current global macadamia cultivation area is estimated at approximately 467.00×10^4^ km^2^ (Yunnan Forestry and Grassland Bureau, 2022). However, the combined moderately and highly suitable habitats total 266.80×10^4^ km^2^ (20.05% of the total habitat area). Current cultivation area thus exceeds the combined moderately and highly suitable habitats by 200.20×10^4^ km^2^, indicating potential saturation in optimal zones and expansion into lowly suitable habitats. This pattern suggests future cultivation may increasingly focus on marginally suitable areas.

The total suitable area for *Macadamia* in Brazil has decreased substantially under all scenarios, while the total suitable area for *Macadamia* in China has increased substantially under all scenarios. It can be observed that the suitable area for *Macadamia* in Brazil is projected to retreat to southern high-altitude regions, while the suitable area for *Macadamia* in China is projected to expand northward toward higher latitudes. This is due to global warming, which may cause species to shift their distribution ranges northward or toward higher latitudes (Couet et al., 2022). Species typically tend to migrate to cooler regions (higher elevations/latitudes) to track their climatic niche (Yi et al., 2025).

Regarding habitat dynamics, the largest habitat area in the 2030s occurs under the SSP126 scenario, while the largest habitat areas in the 2050s and 2070s occur under the SSP370 and SSP585 scenarios, respectively. This suggests in near-future periods, lower emissions favor macadamia habitat expansion, whereas in distant-future periods, significant warming under high-emission scenarios may enhance habitat suitability.

### 4.4 Species differences

Only two species within the genus *Macadamia* produce edible nuts and are thus commercially cultivated (Wallace and Walton, 2011). *Macadamia integrifolia* has a native range between 23° and 27°S latitude, whereas that of *M. tetraphylla* is between 27° and 29°S latitude (Trochoulias and Lahav, 1983), indicating that *M. tetraphylla* is inherently adapted to cooler climates. However, our analysis revealed that the min temperature of coldest month (bio6) in highly suitable habitats for *M. integrifolia* ranged from 5.37°C to 10.31°C, whereas for *M. tetraphylla*, it ranged from 6.20°C to 9.67°C. Based on the model predictions, *M. integrifolia* has a greater cold tolerance than *M. tetraphylla*. However, this may be attributed to the geographically broader cultivation of *M. integrifolia*. Growers generally prefer *M. integrifolia* over *M. tetraphylla* owing to its higher and more stable fruit yield, fuller fruits, and superior quality and flavor (Nagao et al., 1992; Li et al., 2024a). Consequently, *M. integrifolia* exhibits greater potential for habitat expansion and agricultural development compared to *M. tetraphylla*.

### 4.5 Potential overlap of suitable areas and sustainable development

Model predictions reveal extensive potential suitable habitats for *Macadamia*, particularly under climate change scenarios, indicating a trend of shift and expansion towards higher latitudes and altitudes. However, while this expansion potential could address global demand, it concurrently poses significant ecological risks. A critical concern is the spatial overlap between these newly identified, particularly low-suitability expansion zones, and globally important biodiversity hotspots. For instance, tropical and subtropical regions-often exhibiting exceptionally high biodiversity-frequently coincide with macadamia’s potential expansion areas (e.g., parts of South America, Southeast Asia, and Africa). Unplanned large-scale plantation establishment in these regions could drive the conversion of natural habitats (e.g., forests, shrublands), leading to habitat loss, landscape fragmentation, and consequent threats to local biodiversity. Therefore, sustainable cultivation practices must be prioritized in macadamia planning and promotion. Key strategies include: (1) Utilizing degraded or marginal agricultural lands for cultivation; (2) Strictly protecting intact ecosystems and biodiversity hotspots, avoiding deforestation for land conversion; (3) Guiding land-use planning using model predictions to proactively avoid ecologically sensitive areas.

## 5 Conclusion

The optimized MaxEnt model and 12 selected environmental factors were utilized to predict the global potential habitats of *Macadamia*. The results indicated that bio1 (annual mean temperature), bio3 (isothermality), bio6 (min temperature of coldest month), bio12 (annual precipitation), and bio14 (precipitation of driest month) were the key bioclimatic factors influencing the *Macadamia* distribution. Highly suitable habitats were defined as areas meeting all of the following criteria: annual mean temperature, 16.25–20.84°C, isothermality 46.01–55.03%, min temperature of coldest month 5.77–10.03°C, annual precipitation 1043.46–2055.65 mm, and precipitation of driest month 31.29–103.00 mm. These ranges of environmental factors represent the optimal conditions for macadamia nut cultivation. Based on these conditions, the most suitable regions for macadamia nut cultivation include the eastern, western, and southern coasts of Australia, southwestern China, southeastern South Africa, southeastern Brazil, eastern Madagascar, northeastern Argentina, and the southeastern and western coasts of the United States. However, under the current cultivation practices, future cultivation is expected to shift toward areas with lower habitat suitability. Climate change projections revealed the following three large shifts in habitat suitability. (1) Under SSP126, the total suitable habitat area expanded consistently from the 2030s to the 2070s. (2) Under SSP245 and SSP370, contraction is expected to occur during the 2030s–2050s, followed by significant expansion in the 2070s. (3) SSP585 conditions promoted expansion during the 2030s–2050s but a sharp contraction in the 2070s. Notably, The potential suitable habitat area for *Macadamia* in Brazil decreased considerably in all scenarios, while the area in China exhibited an increase. From the perspective of spatial dynamics, the areas suitable for *Macadamia* in Brazil and Uruguay have expanded significantly in all periods, while the opposite is true for Myanmar. Furthermore, *M. integrifolia* demonstrated greater expansion potential compared to *M. tetraphylla*. These findings provide valuable theoretical guidance for current macadamia nut cultivation practices and establish a scientific foundation for future optimization and adjustment of the macadamia nut industry.

## Data Availability

The original contributions presented in the study are included in the article/**Supplementary Material**. Further inquiries can be directed to the corresponding authors.
