# Supplementary material for: Global climate change and Macadamia habitat suitability: MaxEnt-based prediction under future scenarios
Source: Front Plant Sci. 2025 Sep 11;16:1658566. doi: 10.3389/fpls.2025.1658566 (PMC12460475; doi:10.3389/fpls.2025.1658566)
Supplement: Supplementary file 1 [file SupplementaryFile1.docx]

Supplementary Material

# Supplementary Tables

Table S1. The 19 bioclimatic variables evaluated and their abbreviations.

| **Abbreviation** | **Bioclimatic variables** |
| --- | --- |
| bio 1* | Annual Mean Temperature |
| bio 2* | Mean Diurnal Range (Mean of monthly (max temp–min temp)) |
| bio 3* | Isothermality (bio2/bio7) (×100) |
| bio 4 | Temperature Seasonality (standard deviation ×100) |
| bio 5 | Max Temperature of Warmest Month |
| bio 6* | Min Temperature of Coldest Month |
| bio 7 | Temperature Annual Range (bio5–bio6) |
| bio 8* | Mean Temperature of Wettest Quarter |
| bio 9 | Mean Temperature of Driest Quarter |
| bio 10 | Mean Temperature of Warmest Quarter |
| bio 11 | Mean Temperature of Coldest Quarter |
| bio 12* | Annual Precipitation |
| bio 13 | Precipitation of Wettest Month |
| bio 14* | Precipitation of Driest Month |
| bio 15* | Precipitation Seasonality (Coefficient of Variation) |
| bio 16 | Precipitation of Wettest Quarter |
| bio 17 | Precipitation of Driest Quarter |
| bio 18* | Precipitation of Warmest Quarter |
| bio 19 | Precipitation of Coldest Quarter |

Note: * represents the bioclimatic variable selected for this study.

Table S2. Contribution of 12 selected bioclimatic variables to the MaxEnt prediction model.

| **Variable** | **Percent contribution (%)** | **Permutation importance (%)** |
| --- | --- | --- |
| bio 3 | 30 | 10.6 |
| bio 12 | 20 | 7.9 |
| bio 14 | 19.6 | 3.8 |
| bio 1 | 10.1 | 22.4 |
| bio 6 | 8.5 | 42.2 |
| slope | 2.5 | 2.3 |
| bio 18 | 2.5 | 2.9 |
| elevation | 2.3 | 0.9 |
| bio 2 | 2 | 2.7 |
| bio 15 | 1.9 | 1.1 |
| bio 8 | 0.8 | 3 |
| aspect | 0.1 | 0.1 |

# Supplementary Figures

**
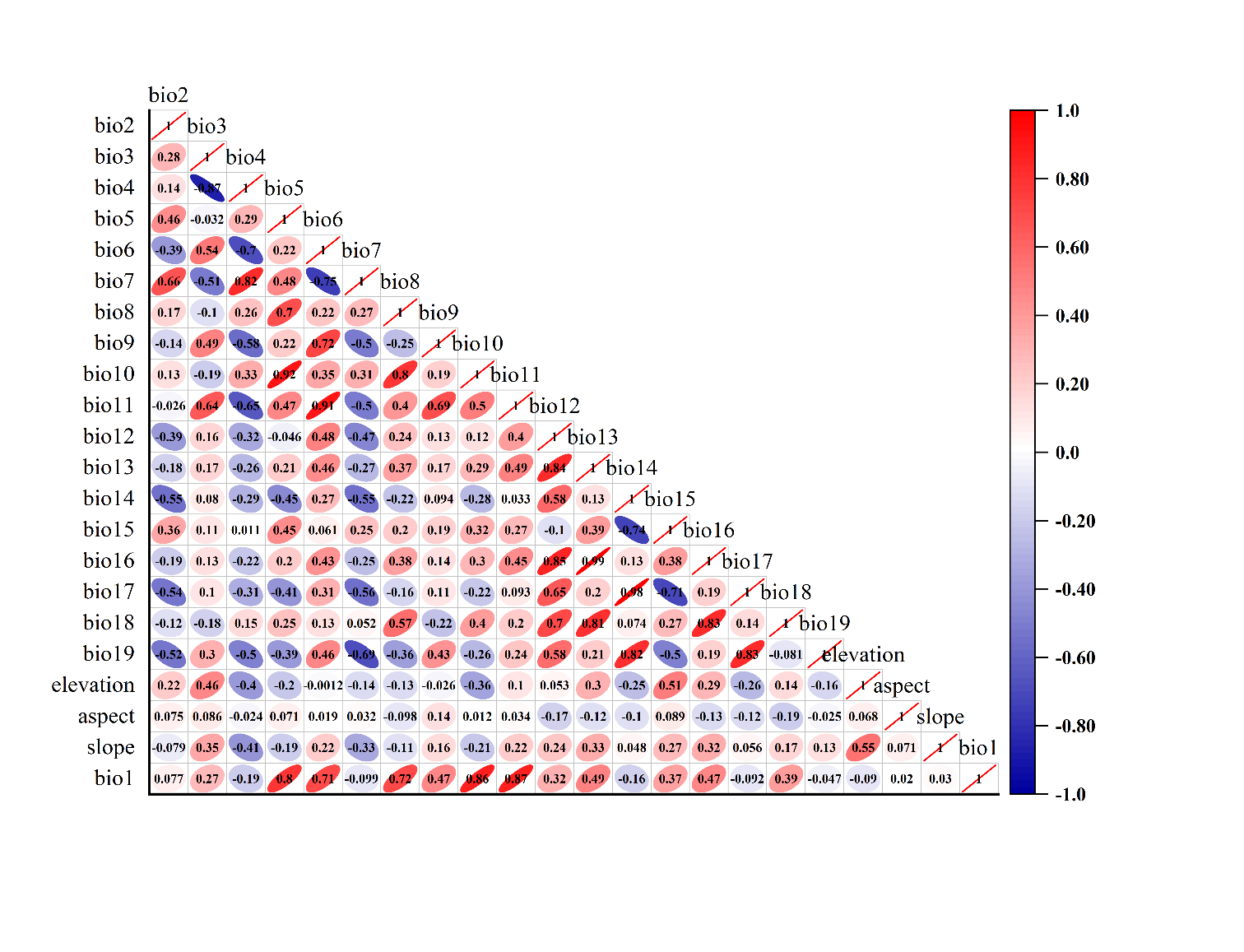
**

Figure S1. Correlation analysis of 19 bioclimatic variables.

Figure S2. Division of *Macadamia* suitable habitats under four future climate scenarios (SSP126, SSP245, SSP370, and SSP585) across three different periods (2030s, 2050s, and 2070s).**
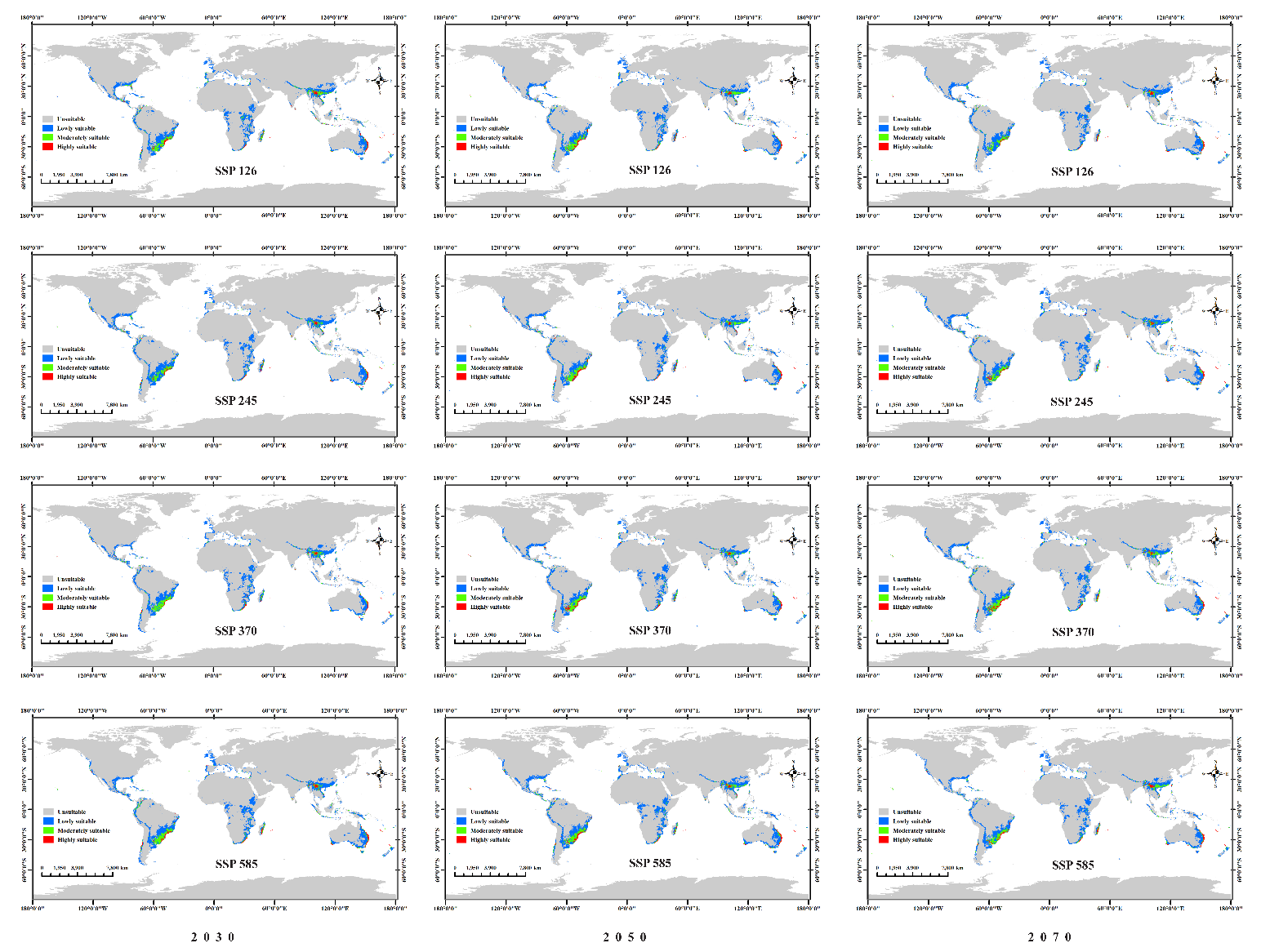
**
